# Characterization of Gastrointestinal Parasites in Grazing Sheep in Mountain, Piedmont, and Savanna Conditions of Casanare, Colombia

**DOI:** 10.1155/vmi/1404541

**Published:** 2026-08-20

**Authors:** José Alfredo Cáceres-Díaz, Ronaldo Tarache-Carreño, Daniela Caro-Estrada, Gloria Estefanía Pastrana-Aguirre, Ciro Ortiz-Valdes, Luis Edgar Tarazona-Manrique

**Affiliations:** ^1^ Veterinary Medicine and Animal Science Program, Faculty of Sciences, Grupo de Investigacion en Ciencias Veterinarias (GINCIVET), Universidad Internacional del Tropico Americano (Unitropico), Yopal, Casanare, Colombia; ^2^ Independent Researcher, Yopal, Casanare, Colombia; ^3^ Faculty of Forestry Engineering, Grupo de Investigacion en Biodiversidad y Dinamica de Ecosistemas Tropicales, Universidad del Tolima, Ibague, Tolima, Colombia, ut.edu.co

**Keywords:** agroecological zones, coinfection, grazing, helminthiasis, parasitic load

## Abstract

Gastrointestinal parasitosis represents a significant constraint on sheep productivity in Colombian tropical production systems; however, their regional epidemiological dynamics remain insufficiently characterized. Therefore, the objective of this study was to characterize the prevalence, associations, coinfestations, and health‐related correlations of gastrointestinal parasites in grazing sheep from the Mountain, Piedmont, and Savanna ecoregions of the Department of Casanare, Colombia. A cross‐sectional observational study was conducted, including quantitative and qualitative coprological examinations, assessments of environmental and production‐related factors, and animal‐level clinical indicators (FAMACHA score and body condition score). Data were analyzed using bivariate analyses (chi‐square or Fisher’s exact test) and nonparametric comparative tests (Mann–Whitney *U* or Kruskal–Wallis test), as appropriate. The overall prevalence was high (78.3%), with higher values in the Mountain ecoregion (90%) compared with the Piedmont (76.7%) and the Savanna (76.1%). A statistically significant association (*p* < 0.05) was identified between municipality, the water source, and parasitic prevalence. Parasitic burden differed significantly among ecoregions (*p* < 0.05). No correlation was observed between animal‐level clinical indicators and parasite burden (*ρ* < 0.065). The distribution of parasite load was aggregated, with a predominance of mild infestations and a small subset of animals harboring burdens exceeding 5000 eggs per gram. Frequent coinfestations among nematodes, protozoa, and cestodes were identified, involving low‐intensity associations. Despite the high prevalence, no clinically relevant health alterations were detected. Gastrointestinal parasitosis exhibited high prevalence, an aggregated distribution of parasite burden, and frequent low‐intensity coinfestations, with variation among ecoregions and no association with clinical indicators. These findings suggest a dynamic process modulated by environmental and management‐related factors, indicating host resilience and highlighting the need for targeted control strategies and longitudinal monitoring.

## 1. Introduction

The global sheep inventory is approximately 1.285 billion head, with China, India, Australia, and New Zealand among the leading producers [1]. Small ruminants constitute valuable sources of meat, milk, wool, horns, bones, and pharmaceutical products; in certain countries, they also play a role in religious practices. Additionally, sheep production is recognized as a low‐cost agricultural activity that contributes to food security, regional economic development, and poverty reduction [2, 3].

Colombia has an inventory of 1,689,875 heads, which supports less than 1% of the Gross Domestic Product (GDP) of livestock, and represents 0.14% of the global population. These animals are primarily concentrated in the Departments of La Guajira (47.35%), Boyacá (13.26%), Cesar (14.38%), Magdalena (8.85%), Cundinamarca (3.34%), Córdoba (2.5%), Santander (1.6%), and Meta (1.6%), with these departments accounting collectively for 92.88% of the national population. In contrast, the Department of Casanare represents only 0.3% of the total sheep inventory [4]. In several Colombian Departments, including La Guajira, Boyacá, and Cesar, as well as in other tropical countries in Latin America and Africa, sheep production constitutes a primary source of livelihood for smallholders in plains, valleys, and mountain areas. Production systems in these regions are characterized by extensive grazing management, small‐scale family‐based operations, low levels of technification, reliance on native pastures, and continued proximity to other livestock species, particularly cattle. Moreover, sanitary management is often irregular, and biosecurity measures are inadequately implemented [3, 5, 6].

These predominant management practices favor the emergence of constraints on productivity and health, including nutritional deficiencies, reduced productivity, reproductive inefficiencies, limited veterinary care, and, consequently, the occurrence of multiple diseases within the flock [3]. Among the most prevalent health disorders, gastrointestinal parasitosis represents the principal problem in small ruminant production systems worldwide. These can be caused by nematodes, trematodes, cestodes, and protozoa, occurring in simple or mixed infestations (coinfections), the latter being more frequent and associated with a greater negative impact than single‐species infestations. Furthermore, the parasitic composition within each host (genera and species involved) and the parasite burden, together with intrinsic factors such as age, physiological status, and breed, as well as extrinsic factors including regional epidemiological and agroecological conditions, determine infestation severity [6, 7]. Consequently, some infestations may remain subclinical while still exerting detrimental effects on flock productivity. In clinical cases, the most common manifestations include reduced productive performance, particularly progressive decreases in weight gain or weight loss (6–12 kg per animal per year), anorexia, and diarrhea. In infestations caused by hematophagous species, anemia and ventral edema may result from loss of blood or plasma proteins, potentially leading to mortality rates exceeding 40% [7, 8].

In Colombia, several studies have identified the most common gastrointestinal parasites affecting sheep production systems; however, these investigations have primarily focused on highly productive departments such as Córdoba [9], Boyacá, and Santander [10–12]. These studies demonstrated that the predominant gastrointestinal parasites in these flocks include protozoa of the family Eimeriidae, nematodes of the family Trichostrongylidae, and trematodes such as *Fasciola hepatica*. Díaz‐Anaya et al. [10] reported parasitic coinfestations in grazing sheep in Boyacá, with biparasitism and triparasitism being the most prevalent patterns. However, these studies did not evaluate the potential influence of regional agroecological characteristics on the results. In a highly biodiverse country such as Colombia, this omission is particularly relevant, as agroecological conditions provide valuable epidemiological insight into parasite dynamics. Environmental factors, including temperature ranges of 18°C–24°C, high and constant precipitation, relative humidity above 80%, soil types, and vegetation cover, favor the maintenance and development of free‐living parasite stages, thereby facilitating their persistence and distribution within flocks [13–15].

This situation, along with the lack of research on this topic in Colombian departments with low sheep inventories, highlights a significant gap in scientific knowledge that, on the one hand, negatively affects farmers’ productive processes and, on the other, impedes the training of veterinary professionals in the regional context. Consequently, it is necessary to undertake epidemiological studies that consider the specific characteristics of each ecoregion and establish the foundation for ecological, epidemiological, and spatial research aimed at comprehensively understanding the dynamics of interactions among these parasitic agents, their hosts, the environment (whether natural or anthropogenically altered), and associated social factors [16–20]. In this context, the development of initial exploratory epidemiological studies on parasitic diagnosis in grazing sheep becomes a priority, contributing to the strengthening of scientific knowledge through an integrative approach and providing the basis for future ecological, epidemiological, and spatial investigations on gastrointestinal parasitism in grazing sheep. Therefore, the objective of the present study was to characterize the prevalence, associations, coinfestations, and health‐related correlations of gastrointestinal parasites in sheep grazing in the Mountain (MT), Piedmont (PM), and Savanna (SV) ecoregions of the Department of Casanare, Colombia.

## 2. Materials and Methods

### 2.1. Informed Consent

Before data and sample collection, farm owners were informed about the study’s objectives, scope, and procedures. Written informed consent was subsequently obtained from each owner before initiating activities on the respective farms. Confidentiality of all collected data was ensured, and authorization for the use of the data for research purposes and for the publication of results was formally granted. This study was conducted in accordance with *Resolución 8430 de 1993* of the Ministerio de Salud de Colombia (Colombian Ministry of Health and Social Prosperity) [21], which classifies this type of research as “minimal risk” because the samples were obtained directly from the rectum using standard veterinary procedures, resulting in minimal, transient discomfort without affecting animal health or welfare. Therefore, formal approval from an Institutional Animal Ethics Committee was not required. Nevertheless, all procedures were carried out in accordance with general animal welfare principles and internationally accepted guidelines for the ethical use of animals in research [21].

### 2.2. Animal Welfare

This study was conducted in accordance with the guidelines outlined in the “Code of Practice for the Care and Handling of Sheep” issued by the National Farm Animal Care Council, to ensure animal welfare throughout the entire handling process [22]. Additionally, sample collection, packaging, labeling, and shipment were performed following the procedures established in the “Manual Veterinario de Toma y Envío de Muestras” (Veterinary Manual for Sample Collection and Submission) of the Pan American Health Organization (PAHO) [23].

### 2.3. Study Design and Population

The study was structured as a descriptive, observational, cross‐sectional epidemiological investigation using a nonprobabilistic sample approach. The sampling design was selected based on the producer’s limited organizational structure, the low perception of sheep production as a formal productive system, and reluctance toward external researchers. Therefore, a snowball sampling strategy was implemented [24, 25]. Initially supported by the departmental sheep and goat production chain, the Department Secretariat of Economic Development, and university students, this approach was used to expand geographical coverage and identify active sheep farms. Using this approach, nine farms with ongoing sheep production were identified, with a total of 166 animals at the time of sampling. On farms with < 30 animals, census sampling was used, whereas for larger herd sizes, simple random sampling was used to select animals within farms.

### 2.4. Study Area: Ecoregion Characteristics and Farm Localization

The study was conducted between June and July 2025, during the rainy season, with cumulative precipitation ranging from 200 to 400 mm, mean temperatures between 24°C and 27°C, and relative humidity between 70% and 90% in the evaluated sectors [26]. Casanare, located in the Colombian Orinoquía region, is characterized by heterogeneous topography, including Andean foothill systems and extensive alluvial plains. The climate is tropical, with a rainy season from April to November and a dry season from December to March. Soils are predominantly acidic and exhibit low natural fertility [27].

**FIGURE 1:**
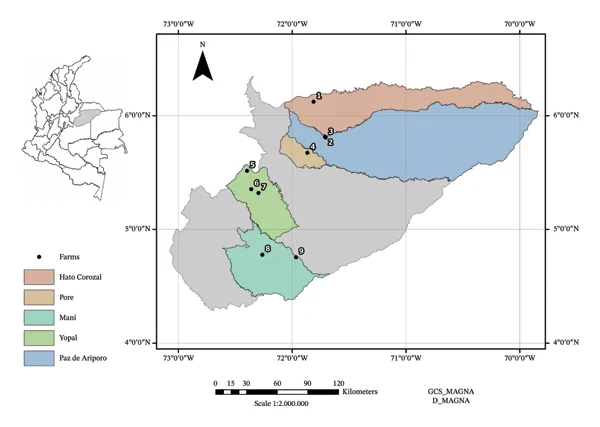
Localization of farms included in the study.

The farms included in the study were distributed across three ecoregions (Figure 1), which, for methodological purposes, were operationally classified as follows:

#### 2.4.1. MT

Corresponding to the eastern slope of the “Cordillera Oriental,” located between 1100 and 4100 m above sea level (m.a.s.l.) [28]. Vegetation cover includes grasslands, herbaceous vegetation, heterogeneous agricultural areas, natural forests, shrublands, and annual or temporary crops [27].

#### 2.4.2. PM

Corresponding to the depositional foothill system of Casanare and Meta, derived from Tertiary and Cretaceous rocks, located between 500 and 1099 m.a.s.l. [28]. Vegetation cover includes grasslands, natural forests, annual or temporary crops, and secondary vegetation [27].

#### 2.4.3. SV

Corresponding to the low floodplains of Arauca and Casanare, located between 0 and 499 m.a.s.l. [28]. Vegetation cover is dominated by grasslands, herbaceous vegetation, and natural forests [27].

The classification of farms into ecoregions was based on altitudinal and agroecological criteria [27, 28]. The proximity observed among some farms (e.g., Farms 2 and 3 in SV) reflects the actual spatial distribution of production systems in the area.

### 2.5. Data Collection and Sampling

Farm‐level characteristics were determined through semistructured interviews with producers to collect information on feeding management, sanitary practices (including the timing of deworming before sampling, the active ingredients used in antiparasitic treatments during the previous 3 months, commercial formulations, dosing schemes, and criteria for administration), water supply, and environmental conditions of the production system. Additionally, individual clinical and productive assessments were performed on each animal to record clinical variables (age, body condition score [BCS], and anemia level) and productive variables, including body weight (BW) (measured using a digital dynamometer), breed (based on phenotypic characterization), and type of water access (classified as running water sources such as rivers or streams, deep wells, or water troughs).

Regarding the production‐related factors and animal‐level clinical indicators, these were determined as follows:

#### 2.5.1. Age

Estimated based on dental eruption patterns and categorized as < 18 months or ≥ 18 months [29].

#### 2.5.2. BCS

Assessed according to the scale described by Romero [29], through palpation of the spinous and transverse processes of the lumbar vertebrae to evaluate bone prominence, muscle mass, and dorsal fat cover. Animals were classified on a five‐point scale: 1 (*emaciated*), 2 (*thin*), 3 (*normal*), 4 (*fat*), and 5 (*obese*).

#### 2.5.3. Breed

Determined through phenotypic characterization with producer support. Animals were classified into three categories: native (Colombian hair sheep), crossbred (individuals without clearly defined purebred phenotypic traits), and introduced breeds (originating from other regions).

Anemia level was determined using the FAMACHA© system [30], which estimates anemia severity based on the color of the lower palpebral mucosa using a five‐point scale, where 1 (*red*) indicates optimal status and 5 (*white*) indicates severe anemia. This method is based on the clinical assessment of anemia, significantly correlated with hematological parameters such as PCV, and is also associated with fecal egg counts (eggs per count [EPG]), particularly in infections dominated by *Haemonchus* spp., supporting its use as an indirect indicator of parasite burden in small ruminants [31]. FAMACHA scoring was independently performed by two previously trained researchers. After individual assessment, results were compared, and discrepancies were resolved by consensus to obtain a final score.

### 2.6. Fecal Sampling and Transport

Approximately 15 g of fecal material was collected from each animal directly from the rectal ampulla to prevent environmental contamination and deposited into individually labeled sterile 20‐mL screw‐cap plastic containers. Samples were transported under refrigeration (4°C–6°C) to the Microbiology and Cell Biology Laboratory of the Universidad Internacional del Trópico Americano within 24 h of collection. Upon arrival, samples were stored at 4°C–6°C and processed within a maximum of 48 h postcollection.

### 2.7. Laboratory Processing

Samples were processed using two complementary diagnostic techniques, based on the information registered in [32], with some modifications:

a. Fecal samples were processed using a centrifugal flotation technique. Initially, 3 g of fresh feces was homogenized with sterile distilled water to obtain a semisolid suspension at a 1:10 ratio. The mixture was then filtered through a double layer of gauze to remove coarse particulate material. The filtrate was transferred to a 15‐mL conical tube (Falcon®), and the volume was adjusted with distilled water to approximately 1 cm below the rim of the tube. Samples were centrifuged at 800 × g for 5 min, after which the supernatant was discarded. The sediment was resuspended in a sucrose solution with a specific gravity of 1.33 until a convex meniscus was formed. A coverslip was placed on the meniscus and left undisturbed for 5 min to allow the flotation of eggs and oocysts. The coverslip was then transferred to a glass slide and examined under a light microscope, starting at 4x magnification and continuing with 10x and 40x for morphological identification [31].
b. Parasitic burden was quantified using a modified McMaster technique based on standardized methodologies. Briefly, fecal samples were homogenized in a sucrose flotation solution with a specific gravity of 1.33 to obtain a uniform suspension. An aliquot of this suspension was collected using a wide‐bore pipette and used to fill both chambers of a precalibrated McMaster counting chamber (Two‐Chamber Green Grid McMaster Slide, Chalex LLC, Park City, UT, USA). The chambers were allowed to stand for approximately 5 min to permit flotation and uniform distribution of eggs and oocysts. Subsequently, all eggs/oocysts within the grids of both chambers were counted under a light microscope at 10x magnification. The number of EPG/OPG of feces was calculated using the standard McMaster formula: *EPG* *=* *(total number of eggs counted in both chambers)* *×* *50*, where 50 corresponds to the correction factor derived from the dilution of the sample and the calibrated volume of the counting chamber. The number of EPG/OPG was calculated by multiplying the total count in both chambers by a correction factor of 50, which accounts for the dilution and chamber volume. The analytical sensitivity of the method was therefore 50 EPG/OPG of feces. Parasitic burden was categorized as mild (50–1000 EPG/OPG), moderate (1000–5000 EPG/OPG), and severe (> 5000 EPG/OPG) [31]. All laboratory analyses were performed in duplicate by two independent laboratory technicians, and the final EPG/OPG of feces was calculated as the arithmetic mean of both readings.
c. Fecal samples positive for strongylid‐type eggs were subjected to larval culture to enable genus‐level differentiation. Briefly, 10 g of fecal material was distributed in thin layers in Petri dishes, which were partially covered to allow oxygen exchange, and incubated under controlled environmental conditions at 25°C for 8 days. Samples were maintained at adequate humidity throughout the incubation period by periodically adding distilled water to prevent desiccation and promote the development of third‐stage infective larvae (L3). Larval recovery was performed using the Baermann technique, based on the active migration of larvae from fecal material into water. Recovered larvae were concentrated and examined under a light microscope at 10x and 40x magnification. Identification to genus level was based on a combination of morphological and morphometric characteristics of L3 larvae, including total length, sheath presence, cranial and caudal morphology, and intestinal cell pattern, according to standard taxonomic keys [32, 33]. Given the potential overlap in morphological features among genera, multiple diagnostic criteria were evaluated simultaneously to improve identification accuracy. For each culture, at least 50 larvae were identified, and when possible, up to 100 larvae were examined to improve representativeness. All larval identifications were performed independently by two trained observers to ensure reproducibility and minimize observer bias.

### 2.8. Data Analysis

Data were initially tabulated in Microsoft Excel®. After database construction and verification, descriptive statistical analyses were performed. Absolute and relative frequencies of gastrointestinal parasite presence were calculated, along with the distribution of identified parasite taxa. The distribution of parasite species was also examined in relation to demographic and physical‐health variables. An exploratory bivariate analysis was conducted to examine the association between the dependent variable, “parasite presence” (positive/negative), and the categorical variables of interest. Contingency tables were constructed for each comparison, and associations were assessed using Pearson’s chi‐square test. When test assumptions were not met (i.e., > 20% of expected cell counts < 5), Fisher’s exact test was applied; for larger contingency tables, the Monte Carlo simulation approach was used [34]. The normality of the quantitative variable “parasitic burden” (EPG/OPG) was assessed using the Shapiro–Wilk test. Because the normality assumptions were not met, nonparametric tests were used to compare parasite counts across categories of the independent variable. The Mann–Whitney *U* test (Wilcoxon rank‐sum test) was used for two‐category variables, whereas the Kruskal–Wallis test was applied for variables with more than two categories [35]. Spearman’s rank correlation coefficient was calculated to evaluate associations between clinical variables and parasitic burden. Finally, a weighted network graph was constructed to visualize the frequencies and prevalence of single and mixed infestations. A significance level of *α* = 0.05 was adopted for all statistical analyses. All tests were two‐sided. Statistical analyses were performed using R software (Version 4.5.2; R Core Team, 2024) within the RStudio environment (Version 2025.05.1) [36]. Data processing and descriptive analyses were conducted using the “dplyr” package [37]. Statistical tests were performed using base R functions.

## 3. Results

### 3.1. Characteristics of Farms Included in the Study

The nine farms included in the study were distributed across three ecoregions of Casanare: SV (*n* = 5; 55.6%; Farms 2, 3, 7, 8, and 9), PM (*n* = 2; 22.2%; Farms 1 and 6), and MT (*n* = 2; 22.2%; Farm 5) (Figure 1). In the SV ecoregion, total farm area ranged from 90 to 2000 ha (ha), with land allocated to sheep grazing between 3 and 100 ha, reflecting variability in production scale. In the PM and MT ecoregions, farms were smaller in total size (40–200 ha), with sheep production occupying approximately 60% of the available land. Most systems (77.7%; *n* = 7) operated under extensive grazing with mineral supplementation, whereas 22.2% (*n* = 2) implemented rotational grazing schemes in the SV ecoregion. Regarding water availability, 77.7% of farms (*n* = 7) relied on natural water sources (streams) located in the PM and MT regions, while 22.2% (*n* = 2) used livestock water troughs supplied by deep‐well water in the SV region, where animals did not have access to natural water sources such as streams or creeks. Sheep production was primarily integrated into mixed farming systems: 66.6% of farms (*n* = 6) raised at least two additional livestock species (cattle, horses, pigs, or goats), while only one farm (11.1%) managed sheep production exclusively. None of the farms had a formally established health management plan supervised by a veterinary professional.

88.9% of farms (*n* = 8) reported using anthelmintic treatments, whereas 1 farm (11.1%) did not implement deworming practices. On each farm, only one anthelmintic was used during the 3 months preceding the visit. Ivermectin 1% (44.4%) was the most used product, followed by fenbendazole 10% (33.3%) and doramectin 1% (11.1%). According to the manufacturers’ recommendations (product label), ivermectin and doramectin were reportedly administered subcutaneously at a recommended therapeutic dose of 0.2 mg/kg BW (practical dosage: 1 mL/50 kg BW), whereas fenbendazole was administered orally at a recommended therapeutic dose of 6 mg/kg BW (practical dosage: 3 mL/50 kg BW). However, the total dose per animal was determined by the producers using a visual estimate of BW. In general, anthelmintic use was empirical or based on recommendations from agroinput suppliers, with no standardized protocols for integrated parasite management.

Additionally, it was observed that 100% of the producers who used anthelmintics indicated that treatments were mainly applied to animals with poor body condition, described as “thin” animals. Furthermore, ivermectin and doramectin were primarily used to control ectoparasites (ticks), which were predominantly located in the auricular pavilion, thereby favoring the development of myiasis in these areas. Their use was also reported in cases of podal myiasis, a frequent problem in the region due to high humidity and sheep’s susceptibility to foot‐related diseases.

### 3.2. Demographic and Clinical Characteristics

A total of 166 animals from five municipalities were included in the study. The highest proportion of sheep was recorded in Maní (27.11%), followed by Paz de Ariporo (21.08%), Yopal (18.10%), and Hato Corozal (18.07%), with lower representation in Pore (15.66%). By ecoregion, most animals were in the SV (68.0%), followed by the PM (25.9%) and the MT (6.0%). Phenotypic breed characterization showed predominance of Colombian hair sheep (42.77%), followed by introduced breeds: Katahdin (28.31%), Dorper (6.02%), Blackbelly (5.42%), Santa Inés (3.61%), Mora Colombiana (1.81%), Pelibuey (0.60%), and crossbred animals (11.45%). Females accounted for 63.25% of the animals, exceeding males by 26.5% points. Most animals belonged to the < 18‐month age group (60.84%). In terms of BCS, the moderate category accounted for the largest proportion (43.98%), followed by thin (28.31%), fat (25.9%), and obese (1.8%). According to FAMACHA scoring, most animals were classified as Category 2 (46.39%), followed by Category 3 (33.13%).

### 3.3. Parasitological Prevalences and Distribution

The overall prevalence of gastrointestinal parasitism was 78.3% (130/166), with values ranging from 62.2% to 100% across the categories of the evaluated variables. Prevalence was similar across ecoregions (MT: 90.0%, PM: 76.7%, SV: 77.8%; *p* = 0.64), grazing system (77.1% vs. 84.6%; *p* = 0.40), grazing area (range: 70.0%–89.3%; *p* = 0.09), anthelmintic treatment (range: 67.7%–93.8%; *p* = 0.06), breed (range: 73.7%–82.9%; *p* = 0.42), and sex (82.9% vs. 70.5%; *p* = 0.06). In contrast, prevalence differed across municipalities (*p* = 0.007), with higher values in Hato Corozal (90.0%), Paz de Ariporo (88.6%), and Pore (88.5%) compared with Yopal (70.0%) and Maní (62.2%). Differences were also observed by water source (*p* = 0.001), with higher prevalence in animals using water troughs (90.9%) compared with those with access to river water (70.0%), corresponding to a difference of 20.9% points (Table 1).

**TABLE 1:** Factors associated with gastrointestinal parasitism in sheep according to demographic and clinical characteristics in Casanare, Colombia.

Variable	Category	Parasitized		Nonparasitized		df^∗^	*S* ^∗∗^	*p* ^∗∗∗^
		*n*	%	*n*	%			
Ecoregion	Mountain	9	90.00	1	10.0	2	0.87	0.64
	Piedmont	33	76.74	10	23.26			
	Savanna	88	77.8	25	23.20			

Municipality	Hato Corozal	27	90.00	3	10.0	4	14.23	0.007
	Maní	28	62.22	17	37.88			
	Paz de Ariporo	31	88.57	4	11.14			
	Pore	23	88.46	3	11.54			
	Yopal	21	70.00	9	30.0			

Grazing system	Extensive grazing	108	77.14	32	25.86	1	0.72	0.40
	Rotational grazing	22	84.62	4	15.38			

Grazing area (ha)	< 10	24	70.59	10	29.41	3	6.51	0.09
	10 < 50	21	70.00	9	30.00			
	50 < 100	35	76.06	11	23.94			
	100–200	50	89.29	6	10.71			

Anthelmintic treatment	Doramectin 1% (10 mg/mL)	15	93.75	1	6.25			0.06
	Fenbendazole 10% (100 mg/mL)	42	67.74	20	32.26			
	Ivermectin 1% (10 mg/mL)	64	82.05	14	17.95			
	None	9	90.00	1	10.00			

Water source	Water trough	60	90.91	6	9.09	1	10.23	0.001
	River	70	70.00	30	30.00			

Breed	Creole	53	74.65	18	25.35	2	1.74	0.42
	Introduced	63	82.89	13	17.11			
	Crossbred	14	73.68	5	26.32			

Sex	F	87	82.86	18	17.14	1	3.47	0.06
	M	43	70.49	18	29.51			

Age (months)	< 18	79	78.22	22	21.78	1	0.001	0.97
	≥ 18	51	78.46	14	21.54			

Body condition score (BCS)	Thin	37	78.72	10	21.28	3	5.44	0.14
	Fat	26	81.25	6	18.75			
	Normal	53	72.60	20	27.40			
	Obese	14	100	0	0.0			

FAMACHA	1	24	85.71	4	14.29			0.41
	2	60	77.92	17	22.18			
	3	40	72.73	15	27.37			
	4	6	100	0	0.0			

*Note:* *p* values were obtained using Pearson’s chi‐square test. When expected cell counts were low, Fisher’s exact test was applied, using the Fisher–Freeman–Halton extension for contingency tables larger than 2 × 2.

^∗^df: degree of freedom (reported only for chi‐square tests).

^∗∗^S: statistic value.

^∗∗∗^ *p*: *p* value.

**FIGURE 2:**
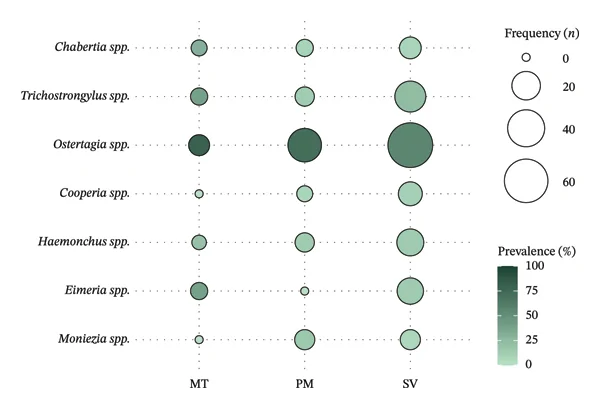
Distribution of parasite prevalence across ecoregions in Casanare, Colombia.

Figure 2 illustrates the percentage distribution of parasite taxa according to the evaluated ecoregions. Circle size and color intensity were jointly used to represent relative frequencies.

A comparable distribution pattern was observed for most parasite taxa across ecoregions. *Ostertagia* spp. showed higher proportions than other identified parasites in all three ecoregions. *Cooperia* spp. and *Moniezia* spp. were not detected in the MT ecoregion.

Representative photomicrographs of the parasitic structures identified during the coproparasitological analyses are provided in Supporting Figure S1. These include strongyle‐type eggs and structures morphologically compatible with cestode eggs observed by flotation, as well as infective third‐stage larvae (L3) morphologically compatible with *Haemonchus* spp. and *Trichostrongylus* spp. recovered through larval culture followed by the Baermann technique.

### 3.4. Distribution of Parasitic Burdens

Parasite egg/oocyst counts showed wide variability across the evaluated categories. Parasite burden (EPG/OPG) is presented as median and interquartile range (P25–P75) due to the non‐normal distribution of the data (Shapiro–Wilk test, *p* < 0.05). Statistically significant differences in parasite burden were observed among ecoregions (*p* = 0.03), with higher median values in the PT (750 EPG/OPG) and MT (700 EPG/OPG) compared with the SV (200 EPG/OPG). Sheep grazing in the SV exhibited a mild parasite load; however, the median values, also mild, of MT and PT exceeded those of the SV by 500 and 550 EPG/OPG, respectively; MT and PM showed higher median ranges for parasite load. However, not statistically significant within‐category variation (*p* > 0.05) in parasite burden (EPG/OPG) was detected for grazing system, grazing area (ha), anthelmintic used, water access, breed, or sex. Descriptively, median values for grazing area categories ranged between 100 and 350 EPG/OPG. Regarding the grazing system, animals under rotational grazing showed median counts 150 EPG/OPG higher than those under extensive grazing. Regarding anthelmintic type, animals treated with 1% doramectin and untreated sheep had the highest median burdens (725 and 700 EPG/OPG, respectively), whereas those treated with 1% ivermectin or 10% fenbendazole had median burdens of 200 EPG/OPG. With respect to water access, animals with access to natural streams had median counts 150 EPG/OPG higher than those using watering troughs. Across breed groups (creole, introduced, and crossbred), the medians ranged from 150 to 200 EPG/OPG. Female sheep showed median parasite counts 50 EPG/OPG higher than males (Table 2).

**TABLE 2:** Comparison of parasite burden (EPG/OPG) across categories of demographic and productive variables in sheep from Casanare, Colombia.

Variable	Category	*n*	Median (P25–P75)	*p* value
Ecoregion	Savanna	113	200 (50–500)	0.03
	Piedmont	43	750 (50–1850)	
	Mountain	10	700 – (75–2563)	

Grazing system	Extensive	140	200 (50–813)	0.27
	Rotational	26	350 (50–625)	

Grazing area (ha)	< 10	34	150 (0–438)	0.11
	10–< 50	30	100 (0–588)	
	50–< 100	46	350 (0–775)	
	100–200	56	250 (50–1388)	

Anthelmintic treatment	Ivermectin 1% (10 mg/mL)	78	200 (50–963)	0.09
	Fenbendazole 10% (100 mg/mL)	62	200 (0–400)	
	Doramectin 1% (10 mg/mL)	16	725 (88–1100)	
	None	10	700 (75–2563)	

Water source	River	100	250 (0–1063)	0.83
	Water trough	66	200 (50–538)	

Breed	Creole	71	200 (0–625)	0.70
	Introduced	47	150 (50–7259)	
	Crossbreed	19	200 (25–975)	

Sex	F	105	250 (50–950)	0.14
	M	61	200 (0–750)	

*Note:* Comparisons were performed using the Mann–Whitney (Wilcoxon rank‐sum) test for variables with two categories. For variables with more than two categories, the Kruskal–Wallis test was applied. Statistical significance was established at *p* < 0.05.

**FIGURE 3:**
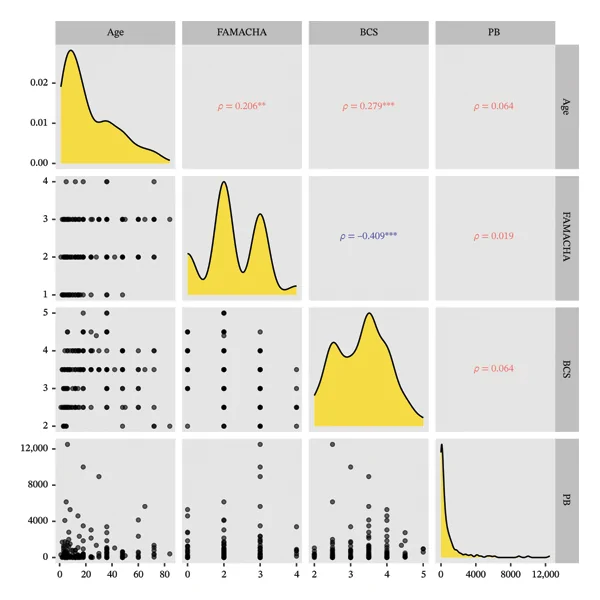
Correlation between health‐related variables and parasite burden (PB) in grazing sheep from Casanare, Colombia. BCS: body condition score. **ρ:** Spearman’s coefficient of correlation.

Correlation analysis between productive and health‐related variables revealed significant associations between age and FAMACHA (*ρ* = 0.206, *p* < 0.50), and between age and BCS (*ρ* = 0.279; *p* < 0.01). A significant association was also observed between FAMACHA and BCS (*ρ* = −0.409; *p* < 0.01). However, parasite egg/oocyst counts showed no significant correlations with the evaluated variables (*ρ* ≤ 0.064, *p* > 0.05), indicating that parasite burden was not directly associated with the productive or clinical characteristics considered in this study (Figure 3).

### 3.5. Characterization of Parasitic Coinfections

**FIGURE 4:**
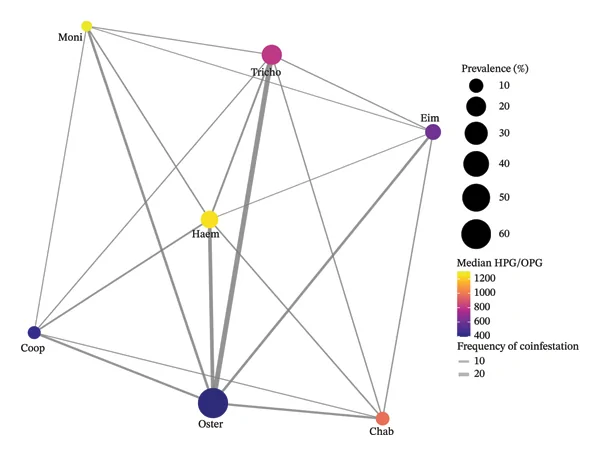
Distribution and prevalence of parasitic burdens by parasite type. Chab: *Chabertia* spp.; Tricho: *Trichostrongylus* spp.; Oster: *Ostertagia* spp.; Coop: *Cooperia* spp.; Haem: *Haemonchus* spp.; Eim: *Eimeria* spp.; Moni: *Moniezia* spp.

Regarding infestation types, single nematode infections accounted for 41.6% of cases, followed by uninfected animals (22.9%). Mixed nematode infections represented 15.7%, while nematode–cestode and nematode–protozoa coinfections accounted for 7.2% and 6.6%, respectively. Protozoa‐only infections reached 4.8%. Less frequent were cestode‐only infestations (0.6%) and multiple coinfections involving nematodes, cestodes, and protozoa (0.6%). Considering parasite burden by parasite type or coinfection, simple nematode infestations, besides being the most frequent, predominantly exhibited low burdens (79.7% with < 1000 EPG), although they contained the highest proportion of severe cases (14.4% with > 5000 EPG). Mixed nematode coinfections mainly showed moderate burdens (50.0% between 1000 and 5000 EPG) with 3.8% high burdens. Nematode–protozoa coinfections (EPG/OPG) had moderate burdens in 36.3% of cases and severe burdens in 1.1% of cases. Coinfections with cestodes and complex combinations (nematodes + cestodes + protozoa) were less common and mostly low‐burden (< 1000 EPG/OPG), while protozoa and cestodes evaluated separately did not show moderate or severe infections. Network analysis of coinfection revealed high interconnectivity among identified parasite genera, indicating an integrated community dynamic rather than independent infections (Figure 4). All parasites showed at least one connection, confirming the frequent coexistence of multiple parasites within the same hosts. *Ostertagia* spp. had the highest structural centrality, with the highest prevalence and the most weighted connections. It also showed an intermediate median parasite burden, suggesting that although widely distributed, its excretion intensity was not the highest among genera. The thickest edges occurred between *Ostertagia* spp. and *Trichostrongylus* spp., as well as between *Ostertagia* spp. and *Haemonchus* spp., reflecting the most common coinfection patterns in the system. In contrast, *Haemonchus* spp. had a relatively high median parasite burden (according to the color scale) but lower prevalence than *Ostertagia* spp., possibly reflecting an epidemiological pattern of lower frequency but higher intensity in affected animals. Less prevalent genera, such as *Moniezia* spp. and *Chabertia* spp., had smaller node sizes but remained connected within the network, indicating their involvement in coinfections, albeit with lower epidemiological weight.

## 4. Discussion

This exploratory study found that the overall prevalence of gastrointestinal parasite infestation in sheep was 78.3%; of these, 71.4% were nematodes, 14.3% were protozoa, and 14.3% were cestodes. Among nematodes, the most frequently detected genera were *Ostertagia* spp*.*, *Trichostrongylus* spp*.*, *Haemonchus* spp., *Chabertia* spp*.*, and *Cooperia* spp., highlighting the widespread distribution of these pathogens in sheep production systems in Casanare. Extensive grazing dynamics may partly explain the low prevalence of protozoa and cestodes, as these parasites are generally more prevalent in enclosed, humid environments where feed and water sources are continuously contaminated with fecal material containing these parasites [15, 19]. These results, combined with the absence of parasites in certain ecoregions (Figure 2) and the statistical association between parasite prevalence and municipalities (Table 1), suggest distinct epidemiological patterns within Casanare. Consequently, control strategies should be tailored to a sequential diagnostic process to establish targeted protocols [38].

The overall prevalence observed in this study is similar to values reported in other regions of the country, particularly in the high tropics; however, differences exist in the types of parasites present. Vargas‐Abella et al. [12] reported an overall prevalence of parasitism of 80%, predominantly *Haemonchus contortus*, across all age groups evaluated at different altitudes in Boyacá. Díaz‐Anaya et al. [10] reported 89.4%, mainly from the families Eimeriidae (63%), followed by Trichostrongylidae (47.4%), Dictyocaulidae (38.1%), and Strongylidae (21.5%), while Pulido‐Medellín et al. [11] found 100% parasite prevalence, primarily coccidian parasites (94.4%), followed by the order Strongylida (33.5%), *Entamoeba coli* (13.3%), *Fasciola hepatica* (7.8%), *Entamoeba histolytica* (4.4%), *Toxocara* spp. (4.4%), *Strongyloides* spp. (3.3%), *Ascaridia* spp. (2.2%), *Giardia* spp. (1.1%), and *Moniezia* spp. (1.1%). Finally, in regions such as the northern Colombian coast, Ensuncho‐Hoyos et al. [9] observed high prevalences of digestive trichostrongylids and *Eimeria* spp. (97.70% and 81.61%, respectively). Collectively, these results show that gastrointestinal parasitism is widespread across the country, and interstudy variability may be explained by agroecological differentiation, which provides diverse biotic and abiotic conditions for the development and proliferation of each parasite species [38].

Regarding infestation types, simple nematode infestations predominated (41.6%), followed by uninfected animals (22.9%) and mixed nematode infestations (15.7%); coinfestations with cestodes (7.2%) and protozoa (6.6%) were less frequent, while exclusive protozoa infestations (4.8%), cestode infestations (0.6%), and multiple combinations (0.6%) were uncommon. In terms of parasitic load, simple nematode infestations were mainly associated with low loads (< 1000 EPG; 79.7%), although they concentrated the highest proportion of severe cases (> 5000 EPG; 14.4%). Nematode coinfestations were mainly moderate, while associations with cestodes and protozoa remained mostly in the low range; evaluated independently, the latter did not show moderate or severe infections. These results are similar to those reported by Díaz‐Anaya et al. [10], where simple infestations were the most frequent, followed by double and triple infestations, and uninfected animals. These findings contrast with those reported by Adhikari et al. [39] and Terfa et al. [3], where polyparasitism was the most common type of infestation; this highlights agroecological differences between studies and calls for longitudinal research in the department to corroborate the pattern observed in this study. Sissay et al. [15] and Terfa et al. [3] found that parasitic infestations are higher during the rainy season and that coinfestations are common, although at a lower proportion than monoinfestations. Mpofu et al. [14] found a statistically significant relationship between gastrointestinal parasite prevalence, mainly nematodes and cestodes, and agroecological regions and climatic seasons, highlighting the rainy season as a potential driver of parasitic problems.

Although statistically significant differences in parasite burden were detected among ecoregions (*p* = 0.03), the observed medians were relatively low, ranging from 150 to 350 EPG/OPG, with some categories reaching values close to 700 EPG/OPG, which are generally considered mild infection levels in sheep. In this context, using these relatively low values to evaluate differences or associations among variables may have limited the ability to detect patterns between groups. Therefore, future studies should consider scenarios with higher parasite pressure or longitudinal approaches to allow a more comprehensive evaluation of these relationships.

Based on the above, a key aspect to determine was the variation of prevalence according to ecoregion, finding higher parasitism in MT sheep compared to PM and SV (90% vs 76.7% and 76.1%) and significant differences in load, with higher medians recorded in areas above 500 m.a.s.l (PM and MT); although the smaller proportion of mountain animals may have influenced this, a distinct epidemiological dynamic cannot be ruled out. These conditions can be explained by considering pastures, grasslands, heterogeneous agricultural areas, natural forests, and shrublands [27], together with well‐drained soils in MT and PT areas [28], which may protect free‐living stages and favor completion of the parasites’ life cycle; additionally, managing animals in smaller grazing areas increases the likelihood of reinfection by concentrating more animals per unit area and grazing previously contaminated pastures. These findings align with eco‐epidemiological studies in Ethiopia, where optimal humidity (80%), temperature < 25°C, rainfall, good soil infiltration, and management primarily favor nematode prevalence, consistent with the observed predominance [3, 40]; furthermore, harsh environmental conditions, malnutrition, pasture deterioration, and continuous use of contaminated areas can induce immunosuppression and increase susceptibility, subsequently leading to higher prevalence, as in the SV ecoregion [3]. Although a clear pattern of prevalence and parasitic load exists in Casanare, it is important to consider that meteorological conditions may modify this prevalence [40]. Sampling was conducted during the established rainy season, with temperatures between 22°C and 26°C and relative humidity of 70%–90%, which are favorable conditions for parasite development [2]. Therefore, variability in parasitic load is expected to vary with the climatic period. In this sense, there is a need for prospective longitudinal studies with sufficient numbers of farms to evaluate seasonal variations in parasitosis across ecoregions.

Sheep drinking from troughs had a 20% higher prevalence than those with access to natural streams; however, this result may have been influenced by the low animal density in the SV and by the inability to confirm exclusive use of each water source, due to potential consumption from temporary puddles. Additionally, temporary puddles formed during rainy periods in pastures may favor parasitic transmission, especially protozoa and trematodes associated with stagnant water contaminated with bovine fecal matter [39, 41–44]. Likewise, trough areas could favor animal concentration and the formation of humid microenvironments around these areas, thereby promoting the survival and transmission of infective forms. Therefore, it is important to determine the role of water consumption points as potential reservoirs of parasitic niches.

On the other hand, although no statistically significant differences were observed, animals for which farmers reported deworming with 1% doramectin showed prevalences and parasitic loads similar to those of animals not dewormed (Tables 1 and 2). Information regarding anthelmintic treatments was obtained through direct contact with producers; it was established that these practices were irregular and not based on sanitary criteria, veterinary advice, or diagnostic laboratory results; also, BW estimation, rather than individual weighing, may have contributed to dosing inaccuracies, potentially resulting in suboptimal drug exposure and reduced effectiveness of parasite control programs under field conditions. Suggesting this as a factor potentially associated with high prevalences and warranting further investigation, these findings are similar to those reported by Adhikari et al. [39], who state that incomplete and irregular protocols may lead to loss of pharmacological efficacy and increased parasite prevalence, as individuals with high parasitic loads are more susceptible to reinfection. Furthermore, incomplete parasite elimination, combined with optimal agroecological conditions for maintaining free‐living stages, as observed in this study, creates an environment conducive to pathogen accumulation in pastures and animals [15, 45, 46].

Despite the high prevalence of parasites observed, no association was found between parasite presence or intensity and the evaluated sanitary variables, including BCS and FAMACHA© (Figure 4). Specifically, animals with higher EPG/OPG counts did not exhibit lower BCS values or higher FAMACHA© scores than animals with lower parasite burdens. The relatively low parasite burdens observed in this study (maximum median count 750 EPG/OPG) may have limited the ability of these indicators to detect differences in infection levels, suggesting that infected animals may maintain homeostasis without evident clinical manifestations at these parasite counts. This observation is consistent with antiparasitic resilience, defined as the ability to sustain productive performance despite infection [38]. The use of BCS and FAMACHA© as indirect indicators of parasite burden within targeted selective treatment (TST) schemes has been widely studied; however, their effectiveness is context‐dependent. Under tropical conditions, BCS has shown greater sensitivity for detecting animals with high parasite burdens, whereas FAMACHA© may fail to identify a substantial proportion of infected animals, particularly when hematophagous parasites are not predominant [47]. Moreover, their diagnostic value varies according to parasite species, production systems, and environmental conditions, limiting their use as universal predictors [48]. Therefore, although associations between BCS, FAMACHA©, and parasite load have been reported under specific conditions, particularly in the presence of *Haemonchus* spp. [31], the lack of association observed in this study may reflect the multifactorial nature of gastrointestinal parasitism in small ruminants.

Also, the low expression of clinical signs could be related to the high proportion of Creole and crossbred sheep evaluated (54.2%), which may be more resilient. In contrast, introduced breeds showed greater variability in parasite loads. This supports the potential of local genotypes to enhance antiparasitic resilience and sustainable productivity [49–51], although further studies are needed to clarify their genetic basis. In this context, identifying circulating parasites and combining indicators such as serial egg counts, anemia assessment, and productive and immunological parameters may provide a more robust basis for decision‐making in sanitary management [38].

Although statistically significant differences in parasite burden were detected among ecoregions (*p* = 0.03), the observed medians were relatively low, ranging from 150 to 350 EPG/OPG, with some categories approaching 700 EPG/OPG, values generally considered mild infection levels in sheep [52]. In this context, the use of these relatively low values may have influenced the ability to detect associations among variables by reducing the contrast between groups [34]. For example, in anthelmintic treatment, differences in median values were observed (200 EPG/OPG for ivermectin and fenbendazole vs. 725 and 700 EPG/OPG for doramectin or no treatment). However, these differences did not reach statistical significance (*p* = 0.09). Therefore, these results should be interpreted with caution, and future studies should consider scenarios with higher parasite pressure or longitudinal designs.

The high proportion of low loads in simple infestations, along with the presence of a subset of animals with > 5000 EPG, reflects this typical aggregated pattern of trichostrongylids such as *Haemonchus contortus* and *Ostertagia* spp., which exhibit high fecundity and adaptive strategies like hypobiosis, favoring abrupt load increases in susceptible hosts [52]. In particular, *Haemonchus contortus* showed a relatively high median load despite a lower prevalence than other genera, indicating a lower frequency but higher intensity epidemiological pattern, consistent with aggregated dynamics described for hematophagous nematodes with high population amplification capacity [53]. This behavior suggests that a small fraction of heavily parasitized animals disproportionately contributes to pasture contamination and transmission, highlighting the importance of targeted control strategies focused on individuals with higher loads as a more efficient measure to reduce infection pressure at the production system level. The observed parasitic coinfection pattern suggests ecological and immunological processes regulating the structure and intensity of parasite communities in hosts.

The low parasitic load observed in mixed infestations is consistent with the typical overdispersion of gastrointestinal nematodes in ruminants, in which most hosts carry low loads [7]. This finding can be interpreted as intraintestinal ecological interactions: competition for anatomical niches (abomasum, small intestine, large intestine) and metabolic resources, and cross‐immune modulation, particularly the shared Th2 lymphocyte response, which restricts massive proliferation of a single dominant species [54]. In nematode–protozoa coinfections, for example, with *Eimeria* spp., the predominance of moderate loads and the low occurrence of severe cases are consistent with the intracellular biology of the protozoa and their low correlation with clinical severity, especially in adult animals with partial immunity [55]. Likewise, the low frequency of moderate or severe loads in coinfections with cestodes such as *Moniezia expansa* is explained by their lower individual pathogenicity and indirect life cycles, which introduce additional ecological constraints that limit their population intensity compared to directly transmitted nematodes [32]. Finally, the participation of less prevalent genera such as *Chabertia* spp., within coinfection networks, although with lower epidemiological weight, confirms partial overlap of gastrointestinal niches and shared environmental exposures during grazing, highlighting that infestation severity depends not only on parasite presence but also on load, number of species involved, differential pathogenic capacity, and ecological and immunological interactions regulating parasite community structure in flocks.

Although the present study was limited by the representativeness of the sample at the departmental and ecoregional scales, particularly in the MT and PM regions, where only two farms were included in each category, the findings provide preliminary evidence of spatial variation in gastrointestinal parasitism. However, the limited number of farms in these ecoregions limits the robustness of interregional comparisons; therefore, these findings should be interpreted with caution and treated as exploratory. In this sense, the results provide relevant empirical evidence for the initial parasitic characterization of the regional sheep production system, constituting a necessary basis for developing further, more extensive investigations [56], especially in territories where the species has received little scientific and productive attention. In epidemiological and ecological studies, designs with moderate or small sample sizes can be informative when they enable the detection of consistent patterns, generate hypotheses, and provide guidance for subsequent confirmatory research, particularly in complex or understudied systems [57].

## 5. Conclusions

The study demonstrated a high overall prevalence of gastrointestinal parasitosis in sheep (78.3%), with higher rates in the MT region (90%) than in the PM (76.7%) and SV (77.8%), and increased parasitic loads in areas above 500 m a.s.l. Parasitic burden showed an age‐related accumulation pattern and a marked overdispersed distribution, with most animals having low loads, and a small proportion (> 5000 EPG) likely contributing disproportionately to pasture contamination. Nematodes were the predominant parasites, occurring mainly as single infections, while coinfections involving nematodes and protozoa or cestodes were less frequent. Despite the high prevalence, no significant clinical alterations were observed, suggesting resilience in the evaluated populations, particularly in Creole and crossbred sheep. These findings highlight the influence of ecological factors such as ecoregion and altitude on parasite dynamics and support the implementation of targeted control strategies focused on high‐burden individuals. For a safe, effective, and efficient sheep production system, integrated parasite management programs should be strengthened to include strategic deworming, monitoring parasite loads, and selecting resilient animals. Furthermore, future studies incorporating molecular diagnostic tools and longitudinal sampling (which were limitations of the study) are recommended to improve the accuracy of parasite identification and epidemiological understanding.

## Author Contributions

José Alfredo Cáceres‐Díaz: conceptualization, methodology, investigation, data curation, and writing–original draft. Ronaldo Tarache‐Carreño: conceptualization, methodology, data curation, and writing–original draft. Daniela Caro‐Estrada: methodology and investigation. Gloria Estefanía Pastrana‐Aguirre: investigation, software, validation, formal analysis, writing–original draft, and writing–review and editing. Ciro Ortiz‐Valdes: conceptualization, methodology, software, validation, formal analysis, investigation, resources, data curation, writing–original draft, writing–review and editing, visualization, supervision, and funding acquisition. Luis Edgar Tarazona‐Manrique: conceptualization, methodology, software, validation, formal analysis, investigation, resources, data curation, writing–original draft, writing–review and editing, visualization, supervision, project administration, and funding acquisition.

## Funding

No funding was received for this manuscript.

## Conflicts of Interest

The authors declare no conflicts of interest.

## Supporting Information

Additional supporting information can be found online in the Supporting Information section.

## Supporting information


**Supporting Information** Supporting Figure 1. Representative images obtained during routine laboratory examination showing the main parasitic structures identified in the study.

## Data Availability

The data that support the findings of this study are available from the corresponding author upon reasonable request.
