# Supplementary material for: Characterization of Gastrointestinal Parasites in Grazing Sheep in Mountain, Piedmont, and Savanna Conditions of Casanare, Colombia
Source: Vet Med Int. 2026 Aug 20;2026:1404541. doi: 10.1155/vmi/1404541 (PMC13494515; doi:10.1155/vmi/1404541)
Supplement: Supplementary file 1 [file VMI-2026-1404541-s001.docx]

Figure S1.

*Representative photomicrographs of parasitic structures identified during coproparasitological examination and larval culture.*

Representative images obtained during routine laboratory examination showing the main parasitic structures identified in the study. (A) Strongyle-type eggs observed by flotation (red arrowheads; 10×). (B) Strongyle-type eggs observed by flotation (40×). (C) Structures morphologically compatible with cestode eggs (red stars; 40×). (D) Infective third-stage larvae (L3) recovered using the Baermann technique (10×). (E) Third-stage larva (L3) morphologically compatible with *Haemonchus* spp. (40×). (F) Third-stage larva (L3) morphologically compatible with *Trichostrongylus* spp. (40×). Representative images are provided for illustrative purposes only and do not constitute a systematic photographic record of all parasitic taxa identified during the study.
